# Fractures of the Forearm

**Published:** 1889-01

**Authors:** John Browning

**Affiliations:** Columbus, Miss.


### FRACTURES OF THE FOREARM.*

BY JOHN BROWNING, M. D., COLUMBUS, MISS.

Believing that we have not attained a perfect method of treating the most common fractures of the forearm, I present for your consideration some new appliances which have been used with good results.

In these fractures it is best to prevent flexion, extension, pronation and supination of the forearm. It is also necessary to keep the forearm splints parallel to each other, transversely and longitudinally.

Colles's and Barton's fractures require a different appliance, which will be described separately.

The roller or bandage has done so much harm in unskilled hands, that I have abandoned its use except on the hand.

With the elbow flexed at a right angle, the inside splint should be a little wider than the forearm, and long enough to extend from behind the elbow to the metacarpo-phalangeal articulation. The outside splint should be of the same width, and long enough to extend from behind the elbow to near the styloid process of the ulna, and should be cut out under the thumb.

Two pieces of the same thickness as the splints, and one inch wide, should be shouldered into the outside and inside forearm splints so as to present a plane surface with them, and secured to the splints with little nails, so that these pieces will be at right angles with the splints, and opposite the axis of the arm, and long enough to extend up the arm to below the prominence of the deltoid muscle.

To these inch-wide pieces are tacked, next the arm above the elbow, two bands of tar-board, wide enough to envelop the arm and meet above the forearm at the elbow as flexed, and extending upward as high as the inch-wide pieces. If higher, they will

*Read before the Southern Surgical and Gynecological Association.

be pushed down by the prominence of the deltoid muscle, and the splints with them, so that the bones will be bent at the point of fracture. The pieces of tarboard should be soaked in hot water so as to be molded to fit the arm, and lined with cotton wadding and cloth next the skin.

The forearm splints should be cut an inch longer than necessary, and after the tar-board bands are tacked on apply the splints and mould the tar-board bands to fit the arm, and then cut the lower end of the splints off the right length.

The forearm splints should be notched on their outer edges, to keep the strips of cloth to be tied around them from slipping.

Strips of cotton wadding should then be laid on the side of the splints next the arm so as to fit the arm, in some places thick, in others thin, and a piece of cloth sewed around the cotton wadding and splints, drawn so tight as to cause the cotton wadding and cloth covering to present a slightly convex surface, as used by the late Dr. F. H. Hamilton on his forearm splints. Too much convexity is not desirable, as the bones may be pushed too far apart, particularly if the strips of cloth are tied too tight around them. There should be but little cotton under the hand and at the elbow.

When the bones are from any cause disposed to separate too far, a many-tailed bandage, applied at the point of separation and extending to the hand, will prevent it.

Apply a narrow roller bandage to the hand from the wrist to near the end of the fingers, apply the splints, and if they fit bind them on with a few strips of cotton cloth, tied around the arm and forearm parts, so as to exercise gentle pressure.

These strips can be loosened or tightened by any one as the arm swells or shrinks. These convex pads serve well to keep the bones apart, but caution is required in their use, as by binding them too tight the bones may be forced too wide apart or pressed out of position.

When the ulna is broken near its middle, the upper fragment is sometimes drawn away from, and the lower towards, the radius, so as to cause a separation of the broken ends. It is at this point that ununited fracture often occurs. To prevent this, a strip of thin board, not quite so wide as the space between the lower edges of the forearm splints when applied, should be padded with cotton and rolled in cotton cloth, and placed underneath the strips which bind the splints on, so as to keep the ulna straight. This thin board should be of the same length as the outside splint.

The forearm should then be placed in a sling, with thin strips of wood sewed in its upper edges.

In 1885 I read a paper before the Mississippi State Medical Association, describing two rectangular splints, which I had used in these fractures, before I had seen Dr. John H. Packard's account of the use of an internal rectangular splint.

Dr. Scott's rectangular splint prevents motion of the elbow, pronation and spination, but does not provide for keeping the bones apart, and the forearm is in a position of forced supination, which is very uncomfortable.

Finding the straight rectangular splints disposed to slip down, I adopted those described above. Very moderate pressure is required, and it is difficult to displace them.

A many-tailed bandage should be tied around the hand and splint to keep the hand up.

### BARTON'S OR COLLES' FRACTURE.

All the fractures at the lower end of the radius, whether transverse or comminuted, or the oblique fracture first described by Dr. Barton, require extension and counter-extension. These cannot be obtained by using the hand as a lever, and the lower end of the ulna as a fulcrum, even when its semi-articulation with the carpus is not destroyed. The displaced fragments cannot be forced into position and kept there by coaptation splints, and if this should be accomplished, the amount of pressure required leaves the wrist and hand stiffened. Fractures near a joint almost always leave the joint stiffened unless early motion is resorted to. This inflammation is almost certain to attack the articulations of the carpus. Internal and external coaptation splints, no matter how shaped, prevent motion, and more or less pressure on the carpus is necesssary. It is important to preserve the proper position of the lower end of the ulna in relation to the carpus. The loss of the styloid process of the ulna is due to the twisting of the carpus with the fragment of the radius attached inward, and it is also drawn to the radial side of the forearm by the contraction of the muscles attached to the hand. This displacement of the lower end of the ulna is more marked when its semi-articulation with the carpus is destroyed. The contraction of the muscles causing this displacement should be resisted by extension and counter-extension. If one grasps the hand and twists it forcibly inward, a good idea can be formed of the manner in which this displacement occurs. If the muscles of the forearm are resisted by extension and counter-extension in the direction of the axis of the radius, there will be a tendency to readjustment of the fragments, and with very little lateral support this can be accomplished.

For this purpose I use a splint composed of an iron bar, sufficiently strong to prevent motion of the elbow, and keep it flexed at a right-angle. The bar extends from below the prominence of the deltoid muscle to the elbow, where it is bent so as not to press on the elbow, thence along the radial side of the forearm, which is betwen pronation and supination, the thumb being upward, to beyond the end of the first phalanges, the hand being flexed, where it is bent downward at a right-angle about three inches. Where the bar passes over the thumb it is bent upward, so as not to press on it. In front of the arm a tar-board splint or band is riveted to the bar, wide enough to extend from the top of the bar to near the fore-arm, and long enough to nearly envelop the arm.

To the fore-arm part of this bar is secured a similar tar-board splint, by two strips of sheet iron, riveted to the tar-board splint and bent loosely over the iron bar, so that the splint can be made to slide up or down on the bar. This tar-board splint should be as wide as the fore-arm on its inside and a little over half as wide on the outside of the fore-arm, care being taken that it is not wide enough to press on the styloid process of the ulna, nor on the ulna. It should be long enough to extend from near the elbow to the lower end of the radius.

The hand should be flexed loosely, the ends of the first, second and third fingers resting easily on the ball of the thumb. A roller bandage about an inch and a half wide should be passed around the wrist three times and the end stitched. A broad band should be stitched to the bandage around the wrist on the back of the hand and carried around the flexed fingers and stitched to the bandage on the inside of the hand at the wrist, to retain the fingers in a flexed position. The object of this is to preserve the parallel position of the metacarpal bones, and to prevent their being folded inward upon each other by the extension band described as follows. A belt cut biassed to fit the hand near the carpus, and buckled securely so as not to slip over the hand, is applied. Two rings of brass or other metal are sewed to the lower edge of this belt, one on the outside and the other on the inside of the hand. Extension is obtained by a cord passed first over the bent lower end of the bar, the ends being then passed through the rings and tied over the bent end of the bar. Counter-extension is obtained by the pressure of the tar-board splint against the arm above the elbow.

The tar-board splints should be lined with cotton wadding and cloth next the skin, and secured to the arm and forearm by many tailed bandages, the ends tied together. After the cord is tightened sufficiently, the fore-arm tar-board splint should be slipped on the bar towards the hand, until the lower end reaches near the carpus, and can be made to act as a coaptation splint at the point of fracture, but only gentle pressure is required, the contraction of the fore-arm muscles causing the displacement, being resisted by extension and counter-extension.

Sometimes a narrow roller bandage is required to keep the hand from swelling.

Constant slight motion of the carpus is permitted. When the splint is applied the thumb should be under the iron bar. This can be secured by applying the belt around the hand so that the ring on the back of the hand will be near the thumb, and the one on the palmar side nearer the little finger. This causes the hand to be twisted outward, which is the reverse of the abnormal position resulting from the fracture.

If greater lateral pressure should be required, it can be obtained by two pieces of thin board placed transversely opposite the point of fracture, one inside and the other outside the forearm, the ends projecting a little, so that cords can be tied around them below and above the forearm. These pieces will press as required on the tar-board splint.

I have only had to resort to this in a case of ununited fracture of eight weeks' standing.

